# Can ovarian cancer screening work? A secondary analysis of the UK collaborative trial of ovarian cancer screening

**DOI:** 10.1038/s41416-026-03490-2

**Published:** 2026-06-13

**Authors:** Jane M. Lange, Ishfaq Ahmad, Isabel J. Dengos, Andy Ryan, Sophia Apostolidou, Aleksandra Gentry-Maharaj, Shannon Holloway, Marc D. Ryser, Usha Menon, Ruth Etzioni

**Affiliations:** 1 Cancer Early Detection Advanced Research Center, Oregon Health and Science University, Oregon, OR USA; 2 UCL Innovative Clinical Trials Unit, Institute of Clinical Trials and Methodology, University College London (UCL), London, UK; 3 Department of Women’s Cancer, EGA Institute for Women’s Health, University College London (UCL), London, UK; 4 Department of Population Health Sciences, Duke University, Durham, NC USA; 5 Department of Medicine, University of Geneva, Geneva, Switzerland; 6 Division of Public Health Sciences, Fred Hutchinson Cancer Center, Seattle, WA USA; 7 Department of Health Services, University of Washington, Seattle, WA USA

**Keywords:** Cancer screening, Cancer screening, Diagnostic markers

## Abstract

**Background:**

The UK Collaborative Trial of Ovarian Cancer Screening (UKCTOCS; 2001–2020) showed no reduction in disease mortality in its primary intervention arm using multimodal screening (MMS) with longitudinal Cancer Antigen 125 (CA125) and transvaginal ultrasound. Whether this null result reflects the stop-screen design, screening performance, or ovarian cancer natural history remains unclear.

**Methods:**

Using individual-level screening and diagnosis data from the MMS arm, we estimated ovarian cancer natural history, focusing on high-grade serous ovarian cancer (HGSC), the most common and lethal subtype. We then simulated trial outcomes under (1) continued screening beyond the trial screening interval and (2) high sensitivity for early-stage detection over an extended time period.

**Results:**

The estimated window for detecting early-stage HGSC under MMS was <6 months. Continued screening yielded at most a 15% relative mortality reduction. Achieving a ≥20% mortality reduction required extending the detectable early-stage window to 1 year and attaining ≥70% sensitivity during this period.

**Conclusion:**

Current screening modalities offer a very limited opportunity to intercept HGSC at an early stage. Clinically effective ovarian cancer screening will require first- and second-line tests capable of detecting HGSC substantially earlier in its natural history.

## Introduction

Ovarian cancer is the fifth leading cause of cancer death among women [1]. The high incidence of late-stage diagnosis coupled with markedly better survival for early-stage disease has driven decades of research into early detection, culminating in the UK Collaborative Trial of Ovarian Cancer Screening (UKCTOCS). The trial, which launched in 2001, enrolled 200,000 women and included two screen arms, trans-vaginal ultrasound (TVS) and multimodal screening (MMS) using the serum biomarker CA125 followed by TVS. After a median 11.1 years of follow-up, the primary report [2] showed non-significant mortality risk reductions on the MMS and TVS arms of 15% and 11%. Upon extended followup (median 16.3 years), the corresponding mortality risk reductions on each screen arm were less than 10% [3].

The UKCTOCS results have been broadly interpreted as evidence that ovarian cancer screening is not beneficial. In justifying their 2018 recommendation against ovarian cancer screening in average-risk women [4], the US Preventive Services Task Force cited the trial results alongside those of the Prostate, Lung, Colorectal, and Ovarian (PLCO) Cancer Screening Trial [5], which also showed no reduction in ovarian cancer mortality associated with CA125-based screening. A report on the 2016 FDA guidance against using the tests was titled, “Ovarian Cancer Screening Tests Don’t Pass Muster.” [6] The regulatory and policy recommendations have been accompanied by a range of commentaries questioning the value of ovarian cancer screening [7, 8].

The disappointment about screening for ovarian cancer has coincided with growing excitement about new biomarkers for cancer early detection based on liquid biopsy technologies. The new biomarkers will offer blood-based testing for a range of cancers, including ovarian cancer alone [9] or bundled with other cancers in multi-cancer early detection (MCED) tests. Most new blood tests for cancer early detection are still at early phases of evaluation and remain to be studied in efficacy trials. Understanding why a promising existing biomarker-based test failed to yield a benefit would seem to be important as we launch a new era of studies to evaluate a host of new cancer screening biomarkers. Much ink has been spilled speculating about the reasons for the trial’s findings [10–12]. Whether these were driven by study design decisions, poor screening test performance, characteristics of the disease, or inadequate treatment of early-stage cases has not been fully resolved.

Regarding study design, the UKCTOCS essentially followed a stop-screen design where participants are screened for a limited time and then observed after screening stops. This design has been shown to lead to underestimation of screening benefit [13]. It is not known whether the divergent mortality trend presented in the primary trial report might have been sustained had screening continued beyond 2011.

Regarding test performance, an analysis following the initial testing round was optimistic, particularly with respect to MMS, for which sensitivity was estimated to be 89.4%, and specificity 99.8% [14]. We note, however, that the measure of sensitivity used in this study is a proxy for pre-clinical sensitivity of a screening test [15] and may produce overly optimistic results.

Regarding disease characteristics, secondary analyses of ovarian cancer natural history using both published summary data [16, 17] and individual-level patient histories [18] have suggested that the window of opportunity for early detection in the trial was very short, particularly for high-grade serous cancer (HGSC), which accounts for an estimated 70% of diagnoses and 85% of deaths [19]. It is unclear whether lengthening this window of opportunity, even by a modest amount, might have materially altered the trial results.

Regarding adequacy of treatment, Bast et al [10] note that MMS screening led to a reduction of 24% in the incidence of stage IV diagnoses and an increase of 39% in stage I-II diagnoses [3] and questioned whether inadequacy of systemic treatment for early-stage, screen-detected cases might be behind the absence of a corresponding mortality reduction. Menon et al. [12] concluded that technologies with the ability to detect HGSC earlier might be key to improving mortality benefit, particularly if combined with treatment improvements. While this may provide qualitative guidance for future research efforts, it does not offer specific targets for new screening technologies in terms of disease detectability or performance.

As the largest ovarian cancer screening trial, the UKCTOCS provides a uniquely valuable data resource for interrogating these potential explanations. In this study, we present a secondary analysis of the trial designed not only for this purpose but also for identifying specific directions to improve the efficacy of ovarian cancer early detection. Our analysis leverages a stage-specific natural history model that we have developed to estimate the opportunity for early detection. We estimate the model using individual-level screening and diagnosis histories from the trial. We then use the model to predict whether a continued-screen design would have yielded a different result and explore the implications for the mortality benefit of specific improvements in disease detectability and survivability. We focus on HGSC, which represents a particularly challenging target for early detection. Our analysis highlights the success of the trial in terms of the lessons it offers regarding the biology of HGSC and the guidance it provides regarding ovarian cancer early detection research in the future.

## Methods

### UKCTOCS data

The UKCTOCS was a general population trial of average-risk postmenopausal women aged 50 to 74 years of age with 50,640 (25.0%) randomised to the MMS arm, 50,639 (25.0%) to an ultrasound only arm (USS) and 101,359 (50.0%) to no screening between 2001-2005 [20]. The screening arms offered annual tests through December 31, 2011. Compliance with scheduled screening exams was 78% in the USS arm and 81% in the MMS arm. Initial follow-up ended on December 31, 2014, and extended follow-up ended on June 30, 2020.

The MMS arm implemented annual screening using the Risk of Ovarian Cancer Algorithm (ROCA) [21, 22], which assesses an individual’s likelihood of ovarian cancer based on her sequence of CA125 measurements. High- and intermediate-risk ROCA scores led to repeat tests, and if confirmed, to ultrasound. Persistent abnormal findings were followed by a referral for clinical assessment and then to biopsy or surgery, which usually involved laparoscopic removal of tubes and ovaries.

We sourced individual-level data on screening histories (age at and result of each screen); age, mode (screen- or interval detected), and type (HGSC or non-HGSC) of ovarian cancer diagnosis; and age and status at last follow-up (died of ovarian cancer, died of other causes, or censored). Access to the UKCTOCS data was via the UCL Data Safe Haven, a secure computing environment for storing and analysing sensitive data.

To create the analytic dataset, we excluded women who were assigned to a screening arm but did not undergo screening; and we censored women at the time of an abnormal screening outcome other than ovarian cancer, following non ovarian cancer histology at trial surgery or at diagnosis of non-HGSC. We defined early stage as AJCC-7 [23] stage I or II and late stage as III or IV. The 1% of HGSCs that were unstaged were assigned to early stage. In the screen arm HGSC diagnoses and person years at risk were calculated by screen number and year of follow-up after the last screen. In the control arm, diagnoses were tabulated by year of age.

### Ethics approval and consent to participate

All methods were performed in accordance with the relevant guidelines and regulations. The UKCTOCS trial was registered with ISRCTN (ISRCTN22488978) and ClinicalTrials.gov (NCT00058032). All trial participants provided written informed consent for participation. The UKCTOCS study received ethical approval from the UCL Research Ethics Committee (reference 2233.005). The current secondary analysis was determined to be exempt by the Oregon Health & Science University Institutional Review Board.

### Natural history model

**Fig. 1:**
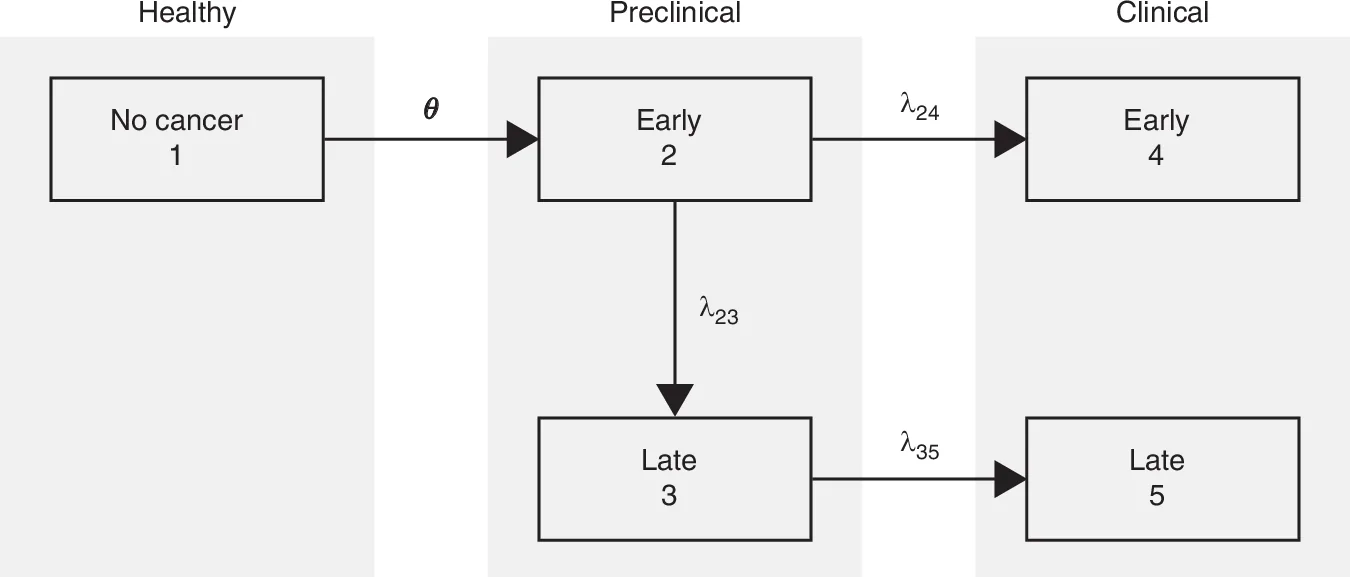
Five-state progressive natural history model.

Our natural history model builds on a classic model of progression between a healthy state and early- and late-stage cancer states [24] (Fig. 1). The “healthy state” encompasses individuals with no cancer, with pre-cancer, or with pre-clinical cancer that is not detectable via screening. The event of transition from the healthy state (state 1) to an early-stage, detectable pre-clinical state (state 2) is termed “pre-clinical detectable onset.” This event occurs when existing diagnostic modalities, here MMS and USS, may detect the disease with some (non-zero) sensitivity. From this point, the tumour may either progress to pre-clinical late stage (state 3) or transition to a clinical or symptomatic state in early stage (state 4). State 5 represents clinically detected late-stage disease.

### Model specifications

For pre-clinical detectable onset, we use a flexible transition rate that varies with age [25, 26] (Appendix A.I, Web Fig. 1). We assume constant transition rates from state 2 (early pre-clinical disease) to 3 (late pre-clinical disease) along with stage-specific sensitivities. We assume a common natural history for individuals in the USS and MMS arms, but allow sensitivities to vary by screening arm. Sensitivity in the model is defined as the probability that the sequence of first and repeat/second line tests identify a cancer if it is present. Lange et al [15] refer to this measure as screening episode sensitivity.

### Model calibration to UKCTOCS data

We estimate the state transition rates and stage-specific sensitivities via maximum likelihood, assuming counts of screen- and interval-detected cases by stage follow a Poisson distribution (Appendix A.II-III). To check the fit of the model, we compare the observed and projected rates of diagnoses over time in the screen and control arms. All statistical analyses are programmed in R 4.4.2 [27]. Model fitting relied on customisation of functions from the R package *cthmm* (https://rdrr.io/rforge/cthmm/).

We also conducted a separate sensitivity analysis using Bayesian calibration with moderately informative priors for the sensitivity parameters (Appendix A.IV).

### Simulating the trial for validation and hypothetical scenarios

**Fig. 2:**
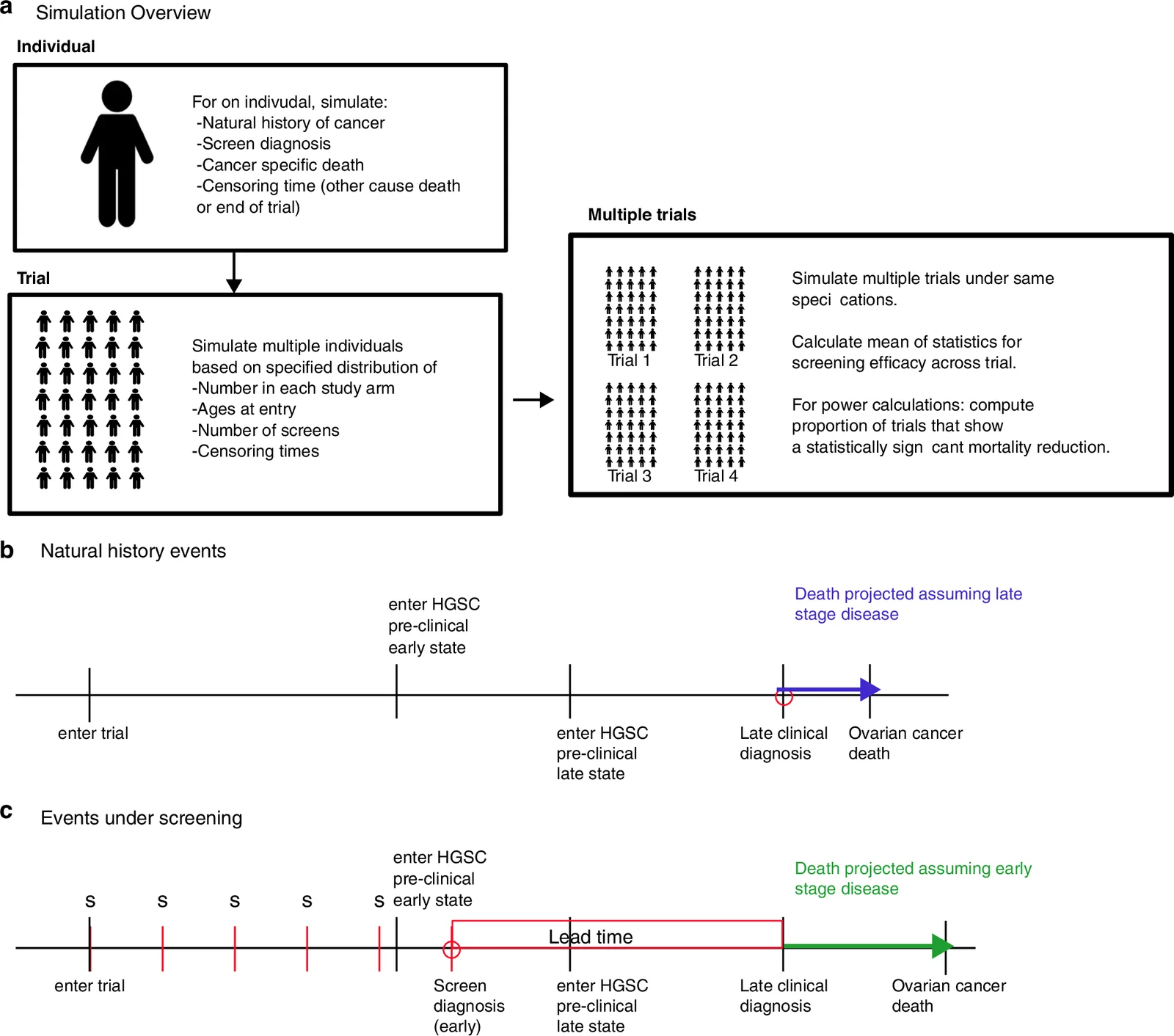
Schematic describing the UKCTOCS simulation. **a** Overview of the trial simulation approach. **b** Example of a woman’s simulated natural history events under no screening. Death is projected assuming late stage at diagnosis. **c** Example of the same woman’s simulated natural history events and time of diagnosis under screening. Given she is detected in an early stage, death is projected post-lead time assuming early stage at diagnosis.

For model validation and investigation of alternative scenarios, we implement repeated simulations (N=400) of the trial given a specified study design and values for state transition rates and screening test performance (Fig. 2A). Each simulation projects diagnoses of and deaths from HGSC over time for 50,000 women representing each of the MMS and TVS arms and 100,000 representing the control arm. Starting ages and numbers of screens are simulated to replicate those in the trial population. To replicate the follow-up time distribution as observed in the trial, we fit a distribution for the time to censoring to the UKCTOCS data by age at entry and screening arm. Then, using the fitted distribution, we simulate an independent time of loss to follow-up or other-cause death.

To project deaths from HGSC without screening, we model HGSC-specific survival as driven by stage at detection. Stage-specific survival times are based on mixture-cure survival models fit to women diagnosed with HGSC in the control arm (Appendix A.V, Web Table 1, Web Fig. 2), estimated with the *cuRe* package [28]. Under screening, cancers detected at an earlier stage receive an increase in their cancer-specific life expectancy based on the difference in survival between early- and late-stage diagnoses without screening. This stage-shift assumption reflects the basic intuition underlying the early detection endeavour and is encoded in the most common mechanistic models of cancer screening benefit [29].

Based on the calibrated natural history model, we simulate individual-level events across each woman’s lifetime, including the age at pre-clinical onset of HGSC, the age at transitions between early and late pre-clinical stages, and age and stage at clinical diagnosis (Fig. 2B). We then superimpose annual screening times and, given test sensitivity, obtain the age at screen diagnosis (Fig. 2C).

To validate the modelled trial outcomes, we compare simulated to observed relative reductions in late-stage diagnosis and HGSC mortality in control and MMS arms. To project results under the scenario of continued screening, we simulate annual screening through 2020. To project results under improved treatment for early-stage cancer, we improve survival for early-stage diagnoses using a hazard ratio of 0.5. To project results of trials under improved disease detectability, we extend the early-stage pre-clinical detectable state to a specified duration (6 months, 1 year, or 1.5 years) under a range of early-stage sensitivities (70–100%). For each specified duration, we re-estimate the model to advance the point of pre-clinical early-stage onset while preserving the distributions of age and stage at diagnosis without screening. We simulate trials under these re-estimated natural history models fixing late-stage sensitivity at the original estimated value.

With the replicate simulation runs, we can estimate uncertainty intervals around the projected outcomes and statistical power. We predict power by calculating the proportion of simulated trials in which the mortality risk ratio or hazard ratio between the control and MMS arms is statistically significant (two-sided $p \, < \, 0.05$) under Cox regression analysis.

## Results

### Descriptive summaries

Table 1 presents summaries based on the 2020 follow-up for the study sample’s MMS(*N* = 50,063), USS(*N* = 48,223) and control (*N* = 101,313) arms, as well as specific results for those diagnosed with HGSC. 1553 HGSCs and 472 non-HGSCs were diagnosed; HGSC constituted 95% of cancer diagnosed in stage III or IV and 95% of cancer deaths. In the MMS arm there was a 12.1% decrease in diagnosed late-stage HGSC and a 6.5% decrease in mortality compared with the control arm. Empirical sensitivity, a proxy for pre-clinical sensitivity [15], measured by the percent of HGSC cancers that are screen detected out of all HGSC cancers diagnosed during the screening interval, was 88.4% (152/172; 95% CI [83.6%, 93.2%]). Analogous outcomes for the USS arm are shown in Table 1. Web Tables 2 and 3 provide counts of screen- and interval-detected cancers by screen number in the MMS and USS arms and counts of cancer detected by age in the control arm.

**Table 1:** Description of sample.

	Control	Multimodal Screening	Ultrasound
A. Full Sample	No. 101,313	No. 50,063	No. 48,223
Age at entry, median (IQR)	60.6 (56.0–66.2)	60.6 (56.1–66.2)	60.5 (56.0–66.0)
Time on study (years), median (IQR)	16.3 (15.1–17.)	16.3 (15.1–17.3)	16.3 (15.1–17.3)
Grade of diagnosis			
No cancer	100,297 (99.0%)	49,549 (99.0%)	47,728 (99.0%)
HGSC	789 (0.8%)	381 (0.8%)	383 (0.8%)
Non-HGSC	227 (0.2%)	133 (0.3%)	112 (0.2%)
HGSC-late stage disease, % change compared to control arm	684	297 (−12.1%)	324 (−0.5%)
HGSC-death, % change compared to control arm	593	274 (−6.5%)	260 (−7.9%)
B. Women diagnosed with HGSC	No. 789	No. 381	No. 383
Stage at diagnosis			
I	40 (5.1%)	43 (11.3%)	28 (7.3%)
II	53 (6.7%)	36 (9.4%)	29 (7.6%)
III	483 (61.2%)	222 (58.3%)	227 (59.3%)
IV	201 (25.5%)	75 (19.7%)	97 (25.3%)
Unable to stage	12 (1.5%)	5 (1.3%)	2 (0.5%)
Mode of diagnosis			
Clinical	789 (100%)	209 (54.9%)	244 (63.7%)
Screen	0 (0%)	152 (39.9%)	81 (21.1%)
Interval	0 (0%)	20 (5.2%)	58 (15.1%)
Empirical sensitivity (screen arms)	NA	88.4%	58.3%

A. Full sample B. Women diagnosed with high grade serous cancer (HGSC). Interval cases are clinically diagnosed within 1 year of a previous screen. Clinical cases are diagnosed after the screening period.

### Fitted model

The natural history model (Web Table 4) estimates a mean early-stage pre-clinical detectable duration of 0.29 years and a mean late-stage pre-clinical detectable duration of 1.5 years (Table 2). The estimated sensitivities were 100% for early- and late-stage HGSC in the MMS arm, but were approximately 50% for the USS arm for both stages. Reliable uncertainty estimates were prohibited due to sensitivity estimates at the boundary of their allowable values. The Bayesian sensitivity analysis (Web Table 5) confirmed a short early-stage pre-clinical duration (posterior mean 0.34, 95% CrI [0.24, 0.59] years) and high sensitivity for pre-clinical disease in the MMS arm (early-stage sensitivity 85.5%, 95% CrI [57.4%, 99.1%]; late-stage sensitivity 91.9%, 95% CrI [77.4%, 99.4%]). Further, in the Bayesian analysis, sensitivity estimates and pre-clinical duration estimates were negatively correlated.

**Table 2:** Calibrated parameter estimates based on fit to UKCTOCS data.

	Maximum likelihood estimate
Mean early stage detectable pre-clinical duration (years)	0.29
Mean late-stage deteectable pre-clinical duration (years)	1.5
Early stage sensitivity (MMS)	100%
Late stage sensitivity (MMS)	100%
Early stage sensitivity (USS)	52%
Late stage sensitivity (USS)	46%

Confidence intervals are not available due to sensitivity estimates being on the boundary of the parameter space.

Graphical goodness-of-fit checks suggested that the model reasonably approximated incidence in the control arm and rates of screen-detected cancers by stage in the screening arms; early-stage interval cancers were modestly overestimated in both arms (Web Fig. 3).

### Validation of model projections against observed outcomes

Using the calibrated model to simulate trial outcomes yielded a close match for the reduction in late-stage HGSC in the MMS versus control arm (Web Fig. 4 and Web Table 5). Simulated mortality hazard ratios were also similar to the observed values.

### Simulated trial outcomes under hypothetical scenarios

Simulations of the trial under the observed stop-screen design with follow-up until 2014 and 2020 suggested low power ($\le \! 20 \%$) to detect a statistically significant mortality benefit on the MMS arm. Continuing screening up to 2020 yielded at most a 15% mortality reduction and power no higher than 49% (Table 3). Improving early-stage treatment yielded an expected mortality hazard ratio of 0.85 (95% CI[0.85, 0.87]) under the 2014 follow-up, modestly better than the projected HR of 0.89 (95% CI [0.89, 0.90]) without improved treatment.

**Table 3:** Summary of simulated hazard ratios and power for detecting mortality reductions in control versus MMS arms under stop- and continued-screen designs.

Design	Year	HGSC Mortality HR [95% CI]	Power [95% CI]
Stop screen	2014	0.89 [0.89, 0.90]	20% [17%, 24%]
	2020	0.93 [0.93, 0.94]	15% [11%, 18%]
Continued Screen	2014	0.85 [0.84, 0.86]	35% [30%, 40%]
	2020	0.86 [0.85, 0.87]	49% [44%, 55%]

*HR* Hazard ratio, *CI* confidence interval.

**Fig. 3:**
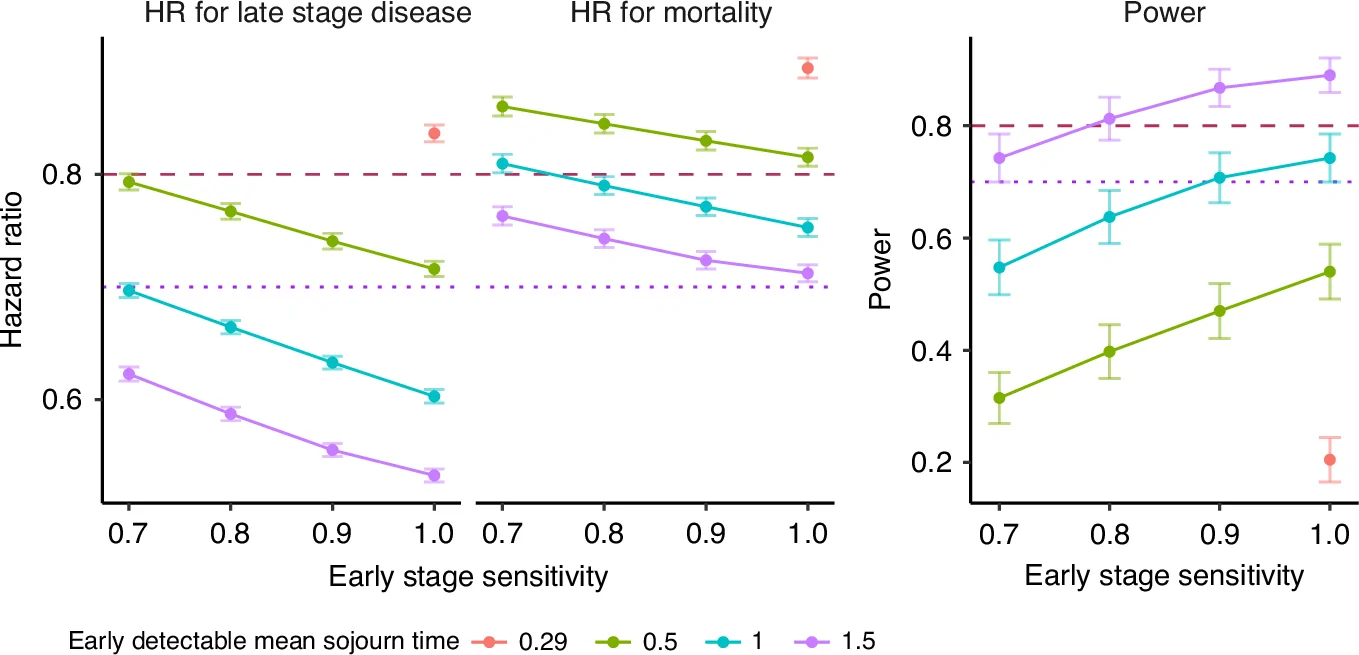
Screening benefit and power of a screening trial under alternative early-stage pre-clinical detection periods. **a** Simulated hazard ratios (mean, 95% CI) for MMS versus control arms for diagnosis of late state HGSC. **b** Simulated hazard ratios (mean, 95% CI) for MMS versus control arms for death from HGSC. **c** Simulated power (mean, 95% CI) of HR of death from HGSC for MMS versus control arms. All are based on the 2014 stop-screen study design.

Figure 3 shows projected trial results extending the early-stage pre-clinical detectable duration and varying early- stage sensitivity. These results suggest an approximately 20% mortality reduction would be expected under an early-stage detectable interval of one year and 70% early-stage sensitivity. With an early-stage detectable interval of 1.5 years, the expected mortality reduction increases to 25%.

## Discussion

Since the publication of the UKCTOCS, disappointment in the results has impacted the entire field of ovarian cancer early detection research. Our secondary analysis leverages the unprecedented opportunity offered by the trial data to infer the natural history of the disease and the performance characteristics of the screening strategies used. We harness the results to model the outcomes of hypothetical what-if scenarios, interrogating in turn different potential explanations for the trial’s failure to show a mortality benefit. In doing so, we derive guidance for future ovarian cancer early detection research efforts.

Our findings enable us to infer with high confidence that the stop-screen design of the trial, while known to lead to attenuated estimates of screening efficacy, was not the explanation for the lack of benefit. We conclude also that inadequate sensitivity of the screening modalities was not behind the negative findings in the MMS arm. Rather, the natural history model shows that the interval of detectability appears to be extremely limited (mean detectable early-stage interval 3–4 months). We conclude that this must be the main explanation for the trial’s null result; when we extend this interval, we project an increase in the expected mortality reduction and in the likelihood that the trial would have produced a statistically significant result.

The limited nature of the opportunity for early detection in the UKCTOCS has been noted by others. Using individual-level UKCTOCS data, Ryser et al. [18] estimated the overall pre-clinical detectable duration of HGSC to be less than 2 years and concluded that the benefit of early detection would be achieved only by prolonging the interception window through judicious combination of first- and second-line tests. Ishizawa et al. [16] also estimated a short pre-clinical detectable duration for HGSC (0.8–1.8 years) based on their analysis of summary data from the UKCTOCS and the PLCO trials.

The limited detectability interval in early stage under the trial’s screening strategies is consistent with our understanding of HGSC biology. Most HGSC tumours are believed to originate in the fallopian tube epithelium as serous tubal intraepithelial carcinoma (STIC) lesions. Wu et al estimated that STIC lesions exist on average 6.5 years before seeding the ovarian surface [30]. Labidi-Galy et al confirmed this time frame, identifying a window of 7 years between development of STIC and ovarian carcinoma, with metastases following rapidly thereafter [31]. These results explain the limited duration of detectable, early-stage disease in the UKCTOCS, which used an imaging strategy on both arms that could not identify tubal lesions, and suggest that it may be possible to extend this interval using a novel second-line testing approach.

Our findings point to the need for screening and confirmation testing technologies that can reliably detect the tubal stage of disease while yielding acceptable positive predictive value (PPV), a challenge in the setting of ovarian cancer screening. The model permits us to explore different settings for performance of both first and second-line tests and devise targets for new biomarkers under development. For example, if the prevalence of early-stage HGSC is 0.5%, then a first-line test with sensitivity 90% and specificity 75%, will yield a negative predictive value (NPV) of 99.9%, and the prevalence of disease will be 1.5% among women with a positive test. This permits ruling out disease with high confidence (important for a first-line test) and yields a test-positive population that is three times enriched for HGSC, reducing the need for extremely high specificity on the second-line test. Now a second-line test with sensitivity of 70% and specificity 95%, will yield an NPV essentially equal to the pre-test probability of having disease (0.5%), and a PPV of 17.6%, leading to 5-6 unnecessary surgeries per cancer detected. The sensitivity of this two-step approach would be 63%; to produce an expected mortality reduction of 20%, these characteristics would need to hold over an interval that is at least 9 months longer than the current early-stage detectable interval. Both first–line and follow-up tests would need to be able to detect disease over this interval. This effectively rules out TVS as a second-line test and opens the door to biomarker-based second-line tests with adequate performance.

Limitations of the model include the constant progression rates between pre-clinical and clinical states and constant stage-specific sensitivities. In practice, sensitivity of a biomarker-based test is expected to improve as disease progresses, but in the setting of the UKCTOCS, where the detectable early-stage interval is short, and sensitivities for both early and late-stage disease are very high, the assumption of constant, stage-specific sensitivity may be reasonable. We assume that all individuals pass through a detectable early-stage state, whereas for some, disease may spread before reaching the limits of detection. These simplifications are needed because pre-clinical durations and test sensitivities must be inferred rather than directly observed and some assumptions and/or constraints are needed to produce unique solutions. We also assume similar natural histories for all three trial arms, but evidence suggests that the detectable pre-clinical duration of HGSC may be modestly longer on the MMS arm than on the USS arm [18]. Despite these limitations, the model reasonably replicates observed patterns of screen and clinical diagnoses, as well as reductions in late-stage disease and mortality.

We conclude that in order to demonstrate clinically significant benefit, screening strategies must extend the period of early tumour detectability. This will require developing credible alternatives for second-line testing to replace TVS. In the future, a two-step approach might use a blood test with high sensitivity to achieve high NPV and triage women with positive results to another blood test with fairly high specificity to achieve high PPV. With adequate performance characteristics over a long enough early pre-clinical duration, this could produce a highly effective strategy for early detection of ovarian cancer.

## Supplementary information


Supplemental Appendix
UKCTOCS Trial Protocol


## Data Availability

De-identified participant data and a data dictionary will be available 12 months after publication. Access requires a methodologically sound proposal, ethical approval, and a signed data access agreement. Proposals should be sent to u.menon@ucl.ac.uk. Following approval and required training, access to data will be provided via the University College London Data Safe Haven.

## Code availability

All code is available at https://github.com/janelange/UKCTOCS.
