# Supplementary material for: Can ovarian cancer screening work? A secondary analysis of the UK collaborative trial of ovarian cancer screening
Source: Br J Cancer. 2026 Jun 13;135(6):890–6. doi: 10.1038/s41416-026-03490-2 (PMC13464898; doi:10.1038/s41416-026-03490-2)
Supplement: Supplementary file 1 [file 41416_2026_3490_MOESM1_ESM.pdf]

Supplementary Materials for "Can Ovarian Cancer Screening Work? A Secondary Analysis of the UK Collaborative Trial of Ovarian Cancer Screening (UKCTOCS)"

Jane Lange PhD[1], Ishfaq Ahmad PhD[1], Isabel Dengos MS[1], Andy Ryan PhD[2], Sophia Apostolidou PhD[2], Aleksandra Gentry-Maharaj PhD[2],Shannon Holloway PhD[3], Marc Ryser PhD[3], Prof Usha Menon FRCOG[2], Ruth Etzioni PhD[4,5]

[1]Cancer Early Detection Advanced Research Center, Oregon Health and Science University,

[2]MRC Clinical Trials Unit, Institute of Clinical Trials &Methodology, University College London

[3]Departments of Mathematics and Population Health Sciences, Duke University

[4]Division of Public Health Sciences, Fred Hutchinson Cancer Center

[5]Department of Health Services, University of Washington

Contents

A. Supplemental Methods **2**

I. Natural history model 2

II. Grouped-level likelihood in a screening trial 4

III. Computing the likelihood 7

IV. Prior specifications for Bayesian natural history model: sensitivity analysis 10

V. Survival curves fit to the control arm 11

Supplementary References **12**

B. Supplementary Tables **13**

1. Weibull mixture-cure parameter estimates 13
2. Observed counts of screen and interval diagnoses 14
3. Observed counts of diagnoses in control arm 15
4. Calibrated natural history model estimates 16
5. Sensitivity analysis: comparison of maximum likelihood and Bayesian estimates of key model parameters 17

C. Supplementary Figures **19**

1. Natural history model with latent onset states 19
2. Weibull mixture-cure models fit to control arm survival data 20
3. Validation of calibrated natural history model against observed incidence of screen, interval, and clinical cancers. 21
4. Comparison of simulated and observed relative cumulative incidence of late- stage disease and mortality 22

A. Supplemental Methods

I. Natural history model

Model states and transition rates

A natural history model is a multistate model that characterizes an individual's transitions throughout disease states of interest. We assume a state space $S = \{1 = \text{no cancer}, 2 = \text{early-stage preclinical HGSC}, 3 = \text{late-stage preclinical HGSC}, 4 = \text{early-stage clinical HGSC}, 5 = \text{late-stage clinical HGSC}\}$, with parameters governing transitions between states as shown in Figure 1. Here, "preclinical" indicates a disease state that is asymptomatic but potentially detectable using a screening test. The term "clinical" indicates a disease state that is symptomatic and detected using established diagnostic tests.

We assume that individuals are at risk of cancer starting at age a_0, and that a measures years since a_0. Let $g_1(a)$ be the age-dependent density of preclinical onset, and let λ_{23}, λ_{24}, and λ_{35} be constant rates of transtionining between states $2 \to 3, 2 \to 4$, and $3 \to 5$, respectively. We use a hypoexponential density for $g_1(a)$, which is equivalent to a series of exponential distributions governed by a set of parameters $\boldsymbol{\theta} = \{\lambda_{1_1 1_2}, \lambda_{1_2 1_3}, \ldots, \lambda_{1_m 2}\}$ (Web Figure 1). These models have a long history in modeling cancer incidence[1] and allow for a flexible onset distribution that increases with age. The dimension of the onset distribution determines the flexibility of the model.

For computational convenience, transitions through the model can be characterized by a latent continuous time Markov chain (CTMC). In a latent CTMC, the progression from healthy to clinical disease states is described by the multi-state process $W(a)$, with state space S, and is based on an underlying time homogeneous CTMC $X(a)$, with state space G.[2] The underlying CTMC specifies constant transition rates between each of the states in G, but the mapping of multiple states in G to S allows for non-constant transition rates between states in S. To translate the model described in Figure 2, we allow for latent states in the preclinical onset distribution only (i.e., the transition from state 1 to 2), resulting in the latent state space $G = \{1_1, \ldots, 1_m, 2, 3, 4, 5\}$, where m is the number of latent preclinical states. The mapping of S into G is:

$$W(a) = 1 \longleftrightarrow X(a) \in \{1_1, \ldots, 1_m\}$$

$$W(a) = 2 \longleftrightarrow X(a) = 2$$

$$W(a) = 3 \longleftrightarrow X(a) = 3$$

$$W(a) = 4 \longleftrightarrow X(a) = 4$$

$$W(a) = 5 \longleftrightarrow X(a) = 5.$$

The underlying CTMC $X(a)$ is parameterized via an intensity matrix $\boldsymbol{\Lambda} = \{\lambda_{ij}\}$, $i, j \in G$ with $i < j$, that describes rates of transitions between latent states and an initial distribution $\boldsymbol{\pi}$. The intensity matrix

is given by:

$$
\mathbf{\Lambda} = \begin{bmatrix}
-\lambda_{1_1 1_2} & \lambda_{1_1 1_2} & 0 & \cdots & & & & & \cdots & 0 \\
0 & -\lambda_{1_2 1_3} & \lambda_{1_2 1_3} & \cdots & & & & & \cdots & 0 \\
\vdots & & \ddots & \ddots & & & & & & \vdots \\
0 & \cdots & & \cdots & -\lambda_{1_{m-1} 1_m} & \lambda_{1_{m-1} 1_m} & 0 & 0 & 0 & 0 \\
0 & \cdots & & \cdots & 0 & -\lambda_{1_m 2} & \lambda_{1_m 2} & 0 & 0 & 0 \\
0 & \cdots & & \cdots & 0 & 0 & -(\lambda_{23} + \lambda_{24}) & \lambda_{23} & \lambda_{24} & 0 \\
0 & \cdots & & \cdots & 0 & 0 & 0 & -\lambda_{35} & 0 & \lambda_{35}
\end{bmatrix}.
$$

Suppose that $\mathbf{P}(t) = \{P_{ij}(t)\}$ is the matrix representing transition probabilities between states i and j in the interval $[s, s+t]$. The time-specific transition probability matrices are characterized by a matrix exponential of the intensity matrix $\mathbf{\Lambda}$:

$$
\mathbf{P}(t) = \exp(\mathbf{\Lambda} t). \tag{1}
$$

Transition probabilities can be computed with standard methods.[3]

II. Grouped-level likelihood in a screening trial

Observed data are grouped by age at entry, number of screens and years of follow-up after a screen. The total person-years at risk and the number of early and late-stage screen or clinical diagnoses are computed by these groupings. The grouped data likelihood is comprised of the likelihood of clinical cases in the control arm ($\mathcal{L}_c$), screen-detected cases in the screen arm $\mathcal{L}_s$, and clinical or interval cases in the screen arm ($\mathcal{L}_{sc}$). The full likelihood for the grouped data is thus

$$\mathcal{L} = \mathcal{L}_c + \mathcal{L}_s + \mathcal{L}_{sc}.$$

To derive the likelihood, we assume that screen, interval, and clinically detected cases have a Poisson distribution with mean equal to the person-years at risk at that screen or interval multiplied by the model-predicted rate of cancers in that screen or interval. The rate is equivalent to the conditional probability of cancer diagnosis in a particular screen or follow-up interval, given being at risk at the screen or start of the interval. We assume censoring is independent of the disease process and thus can be ignored when computing these conditional probabilities.

Notation for grouped-level likelihood

To compute the rates of clinical cancers in the control and screen arms, as well as screen-detected cancers in the screen arm, we calculate the probability of sequences of observed data at specific observation times. We assume that all screens occur at annual intervals. Given the age at entry, a_1, a sequence of observation times is denoted by $\boldsymbol{a} = (a_1, a_2, \ldots, a_n)$, and the observed data by $\boldsymbol{o} = (o_1, o_2, \ldots, o_n)$. Each observation time is associated with a type, indicating whether the observation is a screen or non-screen, denoted by $\boldsymbol{h} = (h_1, \ldots, h_n)$, where $h_i \in \{0 = \text{screen}, 1 = \text{non-screen}\}$.

For a non-screen observation ($h_i = 0$), the data o_i indicate whether early or late clinical cancer is present: $o_i \in \{1 = \text{no clinical cancer}, 4 = \text{early clinical}, 5 = \text{late clinical}\}$. For a screen observation ($h_i = 1$), the data o_i reflects the outcome of the screening episode: $o_i \in \{1 = \text{no cancer}, 2 = \text{early pre-clinical}, 3 = \text{late pre-clinical}, 4 = \text{early clinical}, 5 = \text{late clinical}\}$.

Specific notation and computational details for clinical, screen, and interval-detected cancers are described as follows.

1. **Control arm**: Data are grouped by age at trial entry a_1 and year of follow-up k. For each age at entry, person-years at risk for each year of follow-up are denoted $PY_c(k, a_1)$. $Y_c(j, k|a_1)$ denotes the count of clinically detected cases in stage $j \in \{4, 5\}$, for early or late stage disease, respectively. We denote $r_c(j, k|a_1)$, the probability of clinical cancer in stage j in the interval ($a_2 = k, a_3 = k + 1$) given

an individual is at risk at age a_2. We compute

$$r_c(j,k|a_1) = \frac{P\left(O_1 = 1, O_2 = 1, O_3 = j|\boldsymbol{H} = (0,0,0)\right)}{P\left(O_1 = 1, O_2 = 1|\boldsymbol{H} = (0,0)\right)}.$$

The expected number of cancers diagnosed in stage j is

$$\gamma_c(j,k|a_1) = PY_c(k,a_1) \times r_c(j,k|a_1).$$

The likelihood contribution of the control arm is thus

$$\mathcal{L}_c = \prod_{a1}\prod_{j}\prod_{k} Pois\left(y_c(j,k|a_1); \gamma_c(j,k|a_1)\right).$$

2. **Screen-detected cancers in the screen arm**: Data are grouped by age at trial entry a_1 and screening number ℓ. For each age at entry, the number at risk at a particular screen is denoted $PY_s(\ell, a_1)$, and $y_s(j,\ell|a_1)$ denotes the count of screen-detected cases in stage $j \in \{4,5\}$. We denote $r_s(j,\ell|a_1)$ as the conditional probability of a screen diagnosis at stage j at screen ℓ, given the individual is at risk at screen ℓ. Assume negative screens at a_1, $a_2, \ldots, a_{\ell-1}$ and a screen diagnosis at age a_ℓ. We compute

   $$r_s(j,\ell|a_1) = \frac{P\left(O_1 = 1, O_2 = 1, \ldots, O_{\ell-1} = 1, O_\ell = j|\boldsymbol{H} = (1,1,\ldots,1,1)\right)}{P\left(O_1 = 1, O_2 = 1, \ldots, O_{\ell-1} = 1, O_\ell = 1|\boldsymbol{H} = (1,1,\ldots,1,0)\right)}.$$

   In the denominator, $O_\ell = 1$ and $H_\ell = 1$ indicate that the individual has not had clinical cancer and is still at risk at the time of the screen a_k. The expected number of cancers diagnosed in stage j is

   $$\gamma_s(j,\ell|a_1) = PY_s(\ell,a_1) \times r_s(j,\ell|a_1).$$

   The likelihood contribution of the control arm is thus

   $$\mathcal{L}_c = \prod_{a1}\prod_{j}\prod_{\ell} Pois\left(y_s(j,\ell|a_1); \gamma_s(j,\ell|a_1)\right).$$

3. **Clinically dagnosed cancer in the screen arm**: Data are grouped by age at trial entry a_1 and screening number ℓ and year of follow-up m after a given screen. For each age at entry, the person-years at risk between m and $m+1$ years after a particular screen are denoted $PY_{sc}(\ell, m, a_1)$, and the number of clinical cases in stage $j \in \{4,5\}$ is denoted $y_{sc}(j,\ell,m|a_1)$ We denote $r_{sc}(j,\ell,m|a_1)$ as the probability of clinical diagnosis in the [m, m+1] interval of follow-up after the last screen at age a_ℓ, given a first screen at a_1.

   Interval cancers that occur within one year of a previous screen are denoted $r_{sc}(j,\ell,1|a_1)$. To calculate

this quantity, we assume the individual has negative screens at times $a_1, a_2, \ldots a_\ell$ and has clinical cancer in the interval $(a_\ell, a_{\ell+1} = a_\ell + 1)$. We calculate

$$r_{sc}(j, \ell, 1|a_1) = \frac{P\left(O_1 = 1, O_2 = 1, \ldots, O_\ell = 1, O_{\ell+1} = j|\boldsymbol{H} = (1, 1, \ldots, 1, 0)\right)}{P\left(O_1 = 1, O_2 = 1, \ldots, O_\ell = 1|\boldsymbol{H} = (1, 1, \ldots, 1)\right)}.$$

For clinical cancers diagnosed in the mth interval ($m > 1$), we assume the individual has negative screens at $a_1, a_2, \ldots, a_\ell$, is still at risk at $a_{\ell+1} = a_\ell + m$ and has clinical cancer in the interval $(a_{\ell+1}, a_{\ell+2} = a_{\ell+1} + 1)$. We thus calculate

$$r_{sc}(j, \ell, m|a_1) = \frac{P\left(O_1 = 1, O_2 = 1, \ldots, O_\ell = 1, O_{\ell+1} = 1, O_{\ell+2} = j|\boldsymbol{H} = (1, 1, \ldots, 1, 0, 0)\right)}{P\left(O_1 = 1, O_2 = 1, \ldots, O_\ell = 1, O_{\ell+1} = 1|\boldsymbol{H} = (1, 1, \ldots, 1, 0)\right)}.$$

The expected number of clinical cancers diagnosed in the screen arm in stage j in the mth interval after screen ℓ is thus

$$\gamma_{sc}(j, \ell, m|a_1) = PY_{sc}(\ell, m, a_1) \times r_{sc}(j, \ell, m|a_1).$$

The likelihood contribution of clinically detected cancers in the control arm is thus

$$\mathcal{L}_{sc} = \prod_{a_1} \prod_{j} \prod_{\ell} \prod_{m} Pois\left(y_{sc}(j, \ell, m|a_1); \gamma_{sc}(j, \ell, m|a_1)\right).$$

III. Computing the likelihood

At each observation time, let the underlying latent states of $X(t)$ be denoted by $\boldsymbol{x} = (x_0, \ldots, x_n)$. The observed data $(o_1, \ldots, o_n)$ at each observation time represents a coarsened version of these underlying states. This coarsening arises either from misclassification errors or because multiple latent states in the continuous-time Markov chain (CTMC) correspond to the same observed state. We assume that the misclassification of a given observation is conditionally independent of other observations, given the underlying state at that time. This assumption, combined with the Markov property governing transitions between the latent states $\boldsymbol{x}$, allows the probabilities of observed data sequences of the form

$$P(O_1 = o_1, \ldots, O_k = o_k \mid \boldsymbol{H} = \boldsymbol{h})$$

to be efficiently computed using tools developed for hidden Markov models, such as the forward algorithm.[4]

Components of hidden Markov model

A hidden Markov model is defined by three key components: 1) transition probability matrices that describe the probabilities of transitioning between latent states over time, 2) an emission matrix that gives the probabilities of the observed data conditioned on the latent state at each time point, and 3) an initial distribution that specifies the probabilities of the latent states at the first observation time.

For a given set of observation times $a_i, \ldots a_n$, the transition probability matrices are calculated based on the matrix exponential of $\boldsymbol{\Lambda}$ (Equation 1) for each time interval $a_k - a_{k-1}$, $k \in \{2, 3, \ldots, n\}$,

$$\mathbf{P}(a_k - a_{k-1}).$$

The emission matrix $\boldsymbol{E}(h_k)$ has entries $e(i, j|h_k) = P(O_k = j \mid X_k = i, H_k = h_k)$ for $k \in \{1, 2, \ldots, n\}$, $h_k \in \{0, 1\}$. If there is no misclassification in the observed data, the emission matrix simply maps the observed state space to the state space of $X(a)$. Otherwise, the emission matrix specifies the probabilities of the observed data given the underlying states in $W(a)$, under the assumption that observations are conditionally independent given the underlying state at each observation time. Thus, if k corresponds to a

screening observation ($h_k = 0$), the emission matrix is given by

$$\boldsymbol{E}(h_k = 0) = \begin{array}{c} \begin{matrix} 1 & 2 & 3 & 4 & 5 \end{matrix} \\ \begin{pmatrix} 1 & 0 & 0 & 0 & 0 \\ \vdots & \vdots & \vdots & \vdots & \vdots \\ 1 & 0 & 0 & 0 & 0 \\ 1 - B_e & B_e & 0 & 0 & 0 \\ 1 - B_\ell & 0 & B_\ell & 0 & 0 \\ 0 & 0 & 0 & 1 & 0 \\ 0 & 0 & 0 & 0 & 1 \end{pmatrix} \begin{matrix} 1_1 \\ \\ 1_m \\ 2 \\ 3 \\ 4 \\ 5 \end{matrix} \end{array}.$$

For non-screening observations ($h_k = 1$), the emission matrix is given by

$$\boldsymbol{E}(h_k = 1) = \begin{array}{c} \begin{matrix} 1 & 2 & 3 & 4 & 5 \end{matrix} \\ \begin{pmatrix} 1 & 0 & 0 & 0 & 0 \\ \vdots & \vdots & \vdots & \vdots & \vdots \\ 1 & 0 & 0 & 0 & 0 \\ 1 & 0 & 0 & 0 & 0 \\ 1 & 0 & 0 & 0 & 0 \\ 0 & 0 & 0 & 1 & 0 \\ 0 & 0 & 0 & 0 & 1 \end{pmatrix} \begin{matrix} 1_1 \\ \\ 1_m \\ 2 \\ 3 \\ 4 \\ 5 \end{matrix} \end{array}.$$

We denote the initial distribution of $X(a_1)$ at age a_1 by $\boldsymbol{\pi}(a_1)$. Given a criterion for entering the trial is not having had clinical diagnosis prior to a_1, $\pi_j(a_1) = 0$ for $j \in 4, 5$. For $j \in 1_1, \ldots, 1_m, 2, 3$ we have:

$$\pi_j(a_1) = \frac{P_{1_1 j}(a_1 - a_0)}{1 - [P_{1_1 4}(a_1 - a_0) + P_{1_1 5}(a_1 - a_0)]}.$$

Foward algorithm

The forward algorithm uses a recursion to calculate $P(O_1 = o_1, \ldots, O_k = o_k | \boldsymbol{H} = \boldsymbol{h})$. Using the notation $\boldsymbol{x}_{1:k} = (x_1, \ldots, x_k)$, $\boldsymbol{o}_{1:k} = (o_1, \ldots, o_k)$, and $h_{1:k} = (h_1, \ldots, h_k)$ the forward probabilities are defined for some underlying state $u \in G$ and age a_k as

$$\alpha_k(u) = P(\boldsymbol{O}_{1:k} = o_{1:k}, X_k = u | H_{1:k} = h_{1:k}).$$

The forward probabilities are initialized as

$$\alpha_1(u) = P(O_1 = o_1, X_1 = u|h_1) = e(u, o_1|h_1)\pi_{x_1}(a_1),$$

and the recursion for $k = 2, \ldots, n-1$ is

$$\alpha_k(u) = \sum_i \alpha_{k-1}(i)e(u, o_k|h_k)P_{iu}(a_k - a_{k-1})$$

Thus,

$$P(O_1 = o_1, \ldots, O_n = o_n|\boldsymbol{H} = \boldsymbol{h}) = \sum_u \alpha_n(u).$$

IV. Prior specifications for Bayesian natural history model: sensitivity analysis

The Bayesian specification for the natural history history model assumed a Beta(5.6,1.4) prior for early stage MMS sensitivity (mean .80, 95% prior interval (.45,.98)), and a Beta(7.6,1.4) prior for late stage MMS sensitivity (mean .84, 95% prior interval (.56,.99)). For the USS arm, we specified a uniform prior with mean=.5 (95% range of prior belief (.025,.975)). Natural history rate parameters, specified on the log scale, were limited to the domain [-10,10], with flat priors across their domain. Starting values for model parameters were based on their maximum likelihood estimates. The sampler was run for a total of 2000 iterations, with 500 samples for warm-up. The model was fit with Stan.[5]

V. Survival curves fit to the control arm

Weibull mixture-cure models were fit to cancer-specific survival data from clinically diagnosed patients with HGSC in the control arm, stratified by stage (early or late). Time was measured from diagnosis and censoring events included end-of-study and other-cause death. Mixture cure models allow for a fraction of individuals to never experience the event of interest and provide additional flexibility in capturing the survival outcomes. Weibull mixture-cure models have the form

$$S(t) = \pi + (1 - \pi)S_u(t),$$

where π is the cured fraction and

$$S_u(t) = \exp(\theta_1 t^{\theta_2}).$$

The models were fit via the *R* package *cuRe*.[6] Parameter estimates for the UKCTOCS survival data are shown in Web Table 1, and the fitted survival curves are show in Web Figure 4.

Supplementary References

[1] Armitage P, Doll R. The age distribution of cancer and a multi-stage theory of carcinogenesis. British Journal of Cancer. 1954 Mar;8(1):1-12.

[2] Lange JM, Minin VN. Fitting and interpreting continuous-time latent Markov models for panel data. Statistics in Medicine. 2013 Nov;32(26):4581-95. Available from: https://onlinelibrary.wiley.com/doi/10.1002/sim.5861.

[3] Moler C, Van Loan C. Nineteen Dubious Ways to Compute the Exponential of a Matrix, Twenty-Five Years Later. SIAM Review. 2003 Jan;45(1):3-49. Available from: http://epubs.siam.org/doi/10.1137/S00361445024180.

[4] Rabiner LR. A tutorial on hidden Markov models and selected applications in speech recognition. Proceedings of the IEEE. 1989 Feb;77(2):257-86. Available from: http://ieeexplore.ieee.org/document/18626/.

[5] Stan Development Team. Stan Reference Manual; 2025. Available from: https://mc-stan.org/docs/reference-manual/.

[6] Lasse Hjort Jakobsen. cuRe: Parametric cure model estimation; 2023. Available from: https://CRAN.R-project.org/package=cuRe.

B. Supplementary Tables

Web Table 1: Weibull mixture-cure parameter estimates fit to control arm for early and late stage HGSC

	ρ	θ_1	θ_2
**Early**	0.898	0.1	0.983
**Late**	0.478	0.402	1.025

Web Table 2: Observed counts of screen and interval cases diagnosed in the MMS and USS arms.

Arm	Screen number	Screen (early)	Interval (early)	Screen (late)	Interval (late)	N at screen	Person-years in interval
MMS	1	9	0	19	2	50063	49540.00
MMS	2	7	0	20	0	46225	44159.06
MMS	3	3	0	11	6	43912	41622.74
MMS	4	2	1	9	0	41996	40045.09
MMS	5	10	1	13	1	40226	38192.52
MMS	6	7	0	4	2	38482	36683.12
MMS	7	3	0	11	3	35100	32283.72
MMS	8	4	0	6	2	26046	24470.98
MMS	9	1	0	11	2	16750	15882.49
MMS	10	0	0	1	0	7135	6980.72
MMS	11	0	0	1	0	868	863.99
USS	1	4	0	12	12	48223	43804.94
USS	2	1	0	4	5	44708	42053.25
USS	3	1	0	4	5	42296	39399.89
USS	4	4	1	7	8	40312	37600.72
USS	5	3	0	5	5	38461	35685.71
USS	6	1	1	8	10	36452	34208.02
USS	7	5	0	11	1	32680	30234.84
USS	8	0	0	8	5	23513	21402.71
USS	9	0	0	1	1	14733	13716.65
USS	10	2	0	0	4	6006	5822.17
USS	11	0	0	0	0	619	616.76

Web Table 3: Observed counts of clinically diagnosed cancers in the control arm by age and stage at diagnosis.

Age at diagnosis	Person Years	Early stage	Late stage
50-54	53586.55	5	9
55-59	181765.89	11	31
60-64	302567.44	15	105
65-69	372726.82	24	167
70-74	333066.91	21	184
75-79	208865.34	19	116
80-84	97665.06	8	58
85-89	22751.66	2	13
90-94	505.25	0	1

Web Table 4: Calibrated parameter estimates of the natural history model obtained via maximum likelihood estimation

Parameter	Estimate
$\lambda_{1_1,1_2}$	0.0023
$\lambda_{1_2,1_3}$	0.024
$\lambda_{1_3,2}$	0.024
$\lambda_{2,3}$	3.03
$\lambda_{2,4}$	0.40
$\lambda_{3,5}$	0.65

Web Table 5: Sensitivity analysis: comparison of maximum likelihood and Bayesian estimates of key model parameters

**Parameter**	**Maximum Likelihood**	**Bayesian (mean, 95% CrI)**
Mean early-stage preclinical duration	0.29 years	0.34 [0.24, 0.59] years
Mean late-stage preclinical duration	1.5 years	1.70 [1.25, 2.28] years
Early-stage sensitivity (MMS)	100%	85.5% [57.4, 99.1]
Late-stage sensitivity (MMS)	100%	91.9% [77.4, 99.4]
Early-stage sensitivity (ultrasound)	52%	46.7% [24.9, 73.2]
Late-stage sensitivity (ultrasound)	46%	43.6% [28.5, 61.4]

Web Table 6: Comparison of observed vs. simulated hazard ratios (HR) for MMS versus control arm. Abbreviations: HR=Hazard ratio; CI=confidence interval.

Follow-up year	Observed HR	95% CI	Simulation mean	95% CI
**Reduction in late stage disease: Cox regression HR for MMS versus control arm**				
2014	0.89	[0.75, 1.05]	0.84	[0.83, 0.84]
2020	0.88	[0.77, 1.01]	0.88	[0.88, 0.89]
**Reduction in mortality: Cox regression HR for MMS versus control arm**				
2014	0.87	[0.72, 1.06]	0.89	[0.89, 0.90]
2020	0.94	[0.82, 1.09]	0.93	[0.93, 0.94]

C. Supplementary Figures

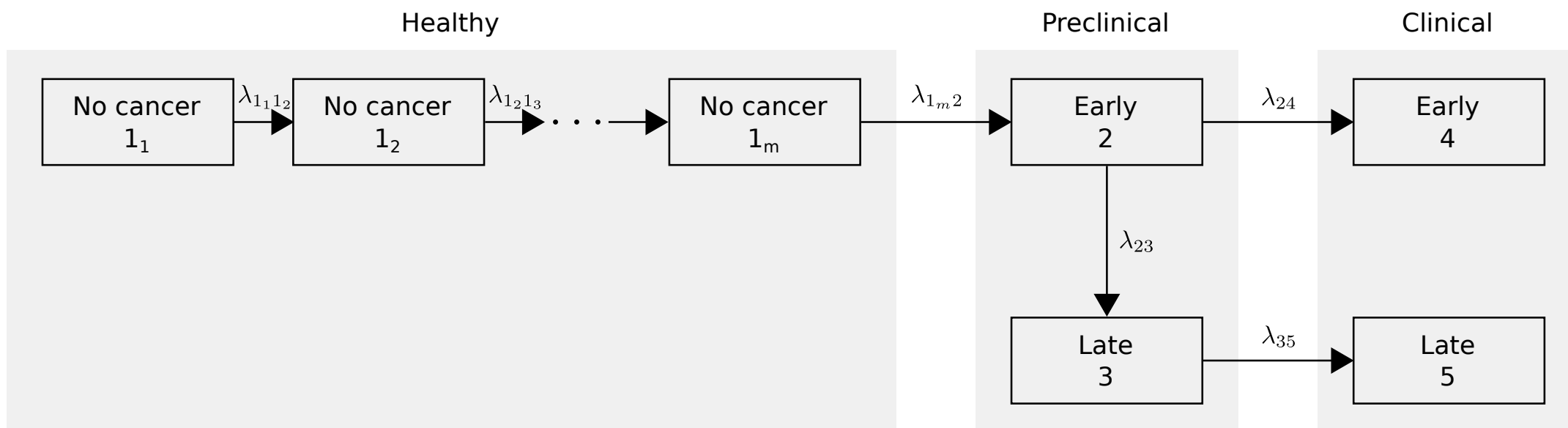

Web Figure 1: Natural history model with hypoexponential preclinical onset distribution.

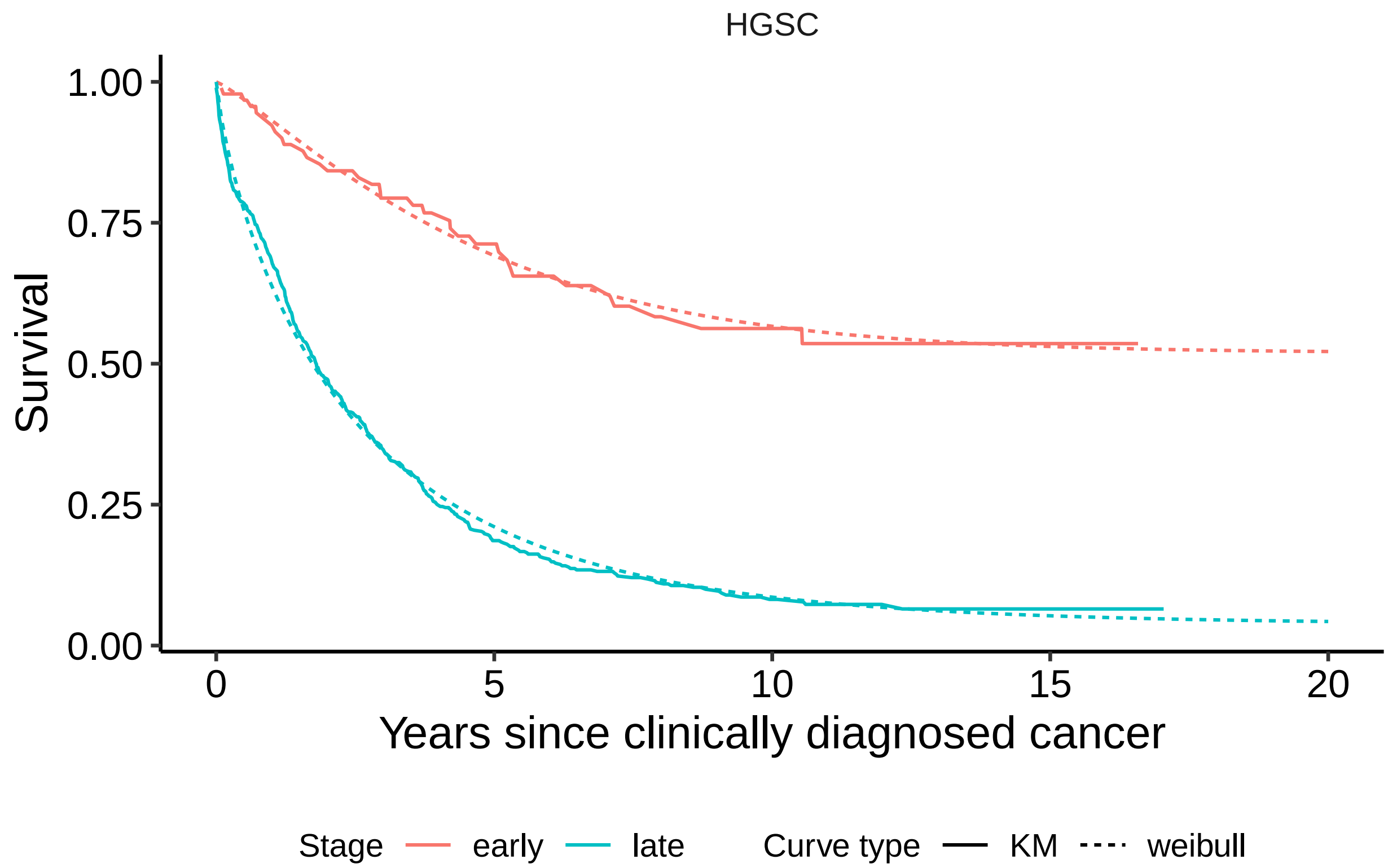

Web Figure 2: Weibull mixture-cure models for cancer-specific survival fit to the control arm for early- and late-stage HGSC.

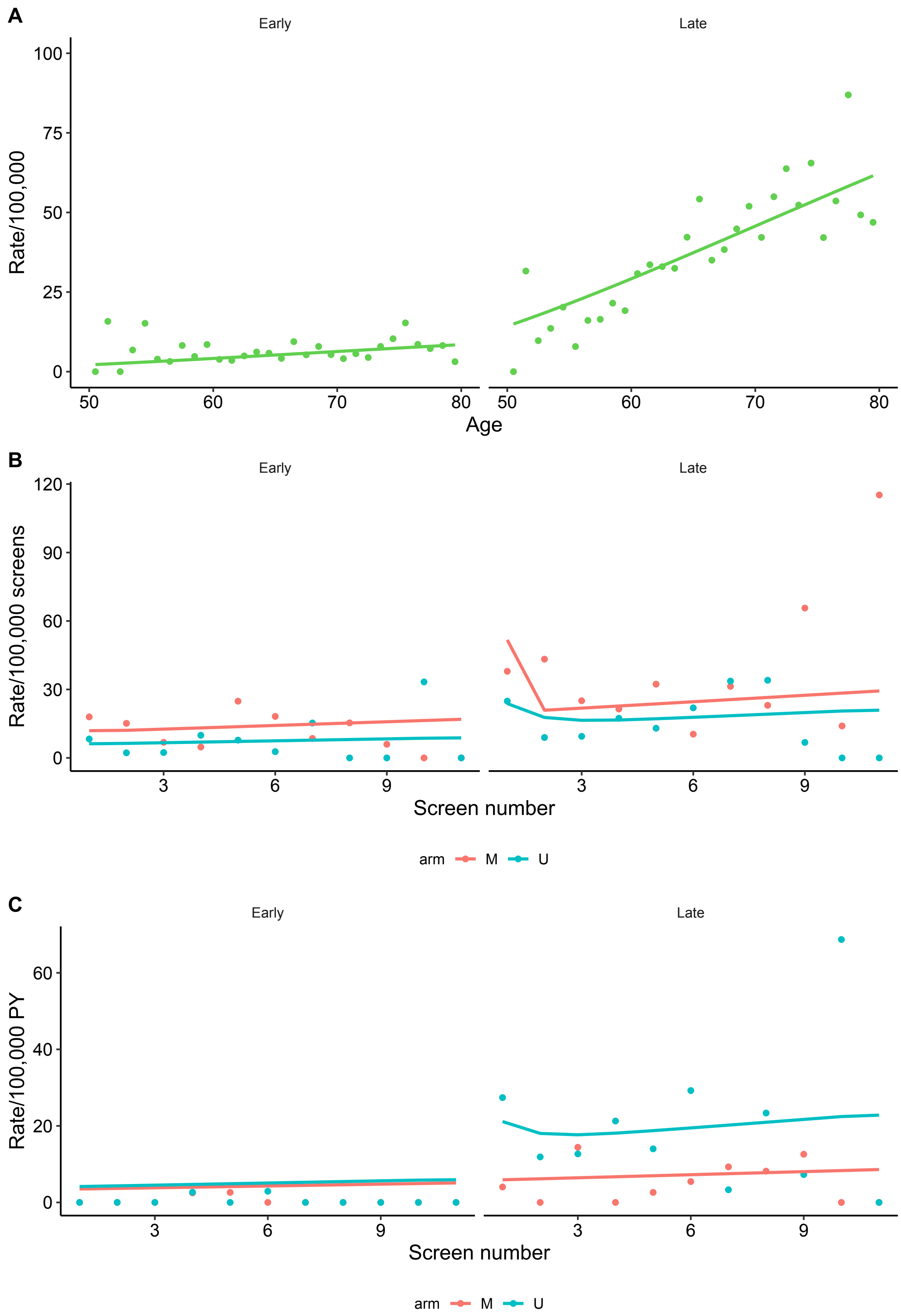

Web Figure 3: Observed versus fitted rates of clinical, screen, and interval high grade serous cancers. A. Clinical cancers in the control arm. B. Screen-detected cancers. C. Interval-detected cancers within 1 year of a previous screen.

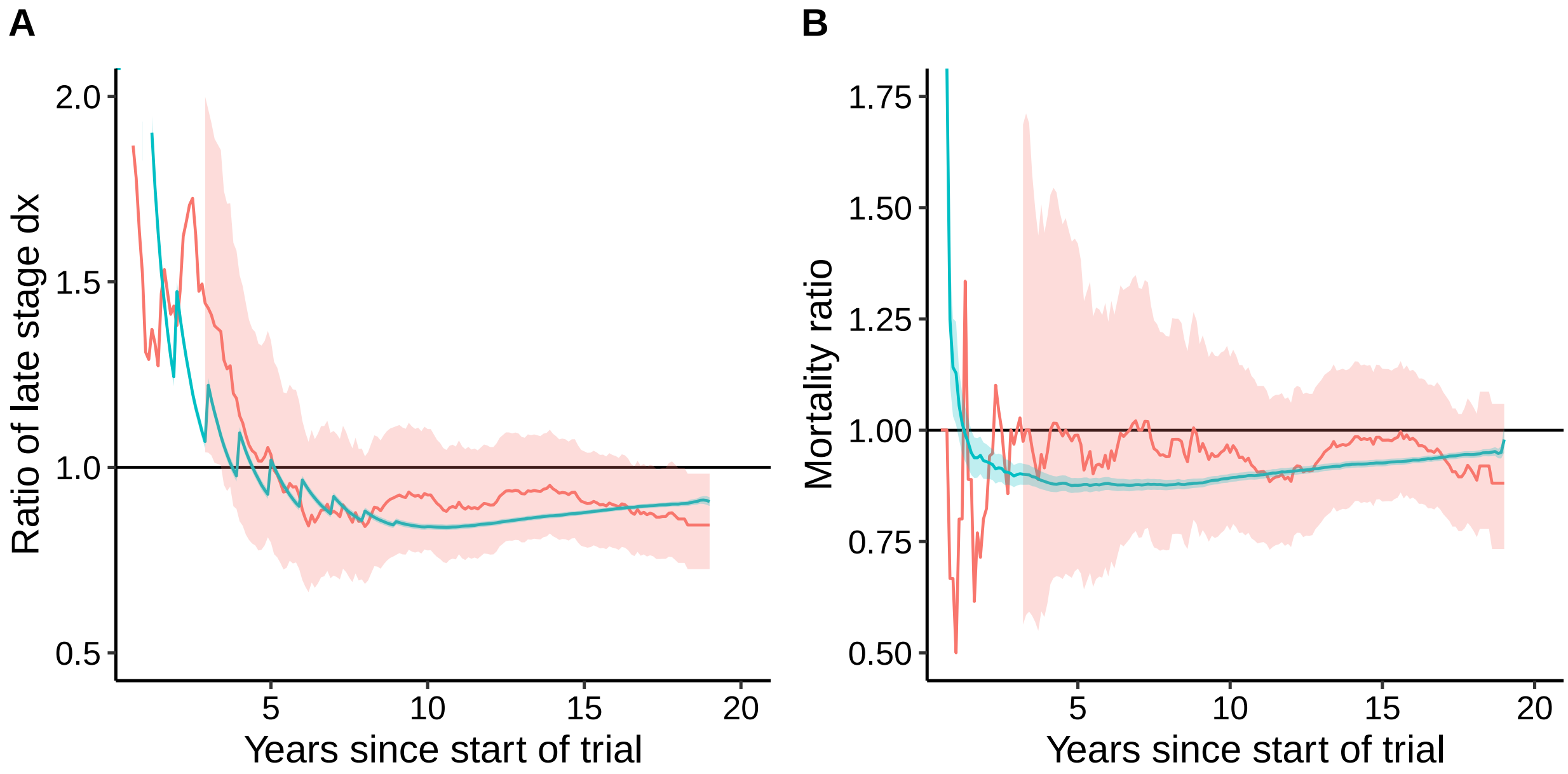

Web Figure 4: Simulated and observed ratios (MMS versus control arm) of A. the cumulative incidence of late stage HGSC B. the cumulative incidence of death due to HGSC. Shading represents 95% confidence intervals.
